# The Culturable Soil Antibiotic Resistome: A Community of Multi-Drug Resistant Bacteria

**DOI:** 10.1371/journal.pone.0065567

**Published:** 2013-06-12

**Authors:** Fiona Walsh, Brion Duffy

**Affiliations:** Bacteriology Research Laboratory, Federal Department of Economic Affairs, Education and Research EAER, Research Station Agroscope Changins-Wädenswil ACW, Wädenswil, Switzerland; U. S. Salinity Lab, United States of America

## Abstract

Understanding the soil bacterial resistome is essential to understanding the evolution and development of antibiotic resistance, and its spread between species and biomes. We have identified and characterized multi-drug resistance (MDR) mechanisms in the culturable soil antibiotic resistome and linked the resistance profiles to bacterial species. We isolated 412 antibiotic resistant bacteria from agricultural, urban and pristine soils. All isolates were multi-drug resistant, of which greater than 80% were resistant to 16–23 antibiotics, comprising almost all classes of antibiotic. The mobile resistance genes investigated, (ESBL, *bla*_NDM-1_, and plasmid mediated quinolone resistance (PMQR) resistance genes) were not responsible for the respective resistance phenotypes nor were they present in the extracted soil DNA. Efflux was demonstrated to play an important role in MDR and many resistance phenotypes. Clinically relevant *Burkholderia* species are intrinsically resistant to ciprofloxacin but the soil *Burkholderia* species were not intrinsically resistant to ciprofloxacin. Using a phenotypic enzyme assay we identified the antibiotic specific inactivation of trimethoprim in 21 bacteria from different soils. The results of this study identified the importance of the efflux mechanism in the soil resistome and variations between the intrinsic resistance profiles of clinical and soil bacteria of the same family.

## Introduction

Antibiotic resistance has developed over time from resistance to single classes of antibiotics to multi-drug resistance and extreme drug resistance. Until recently, antibiotics and antibiotic resistance were discussed in terms of treatments of infections and the prevention of successful treatment, respectively. The mechanisms of action of antibiotics and antibiotic resistance mechanisms have been studied almost exclusively in pathogenic bacteria. It is only in recent years that antibiotic resistance research has focused on the environment from which the antibiotics were initially extracted: soil microorganisms and the soil ecosystem. With an every decreasing supply of novel antibiotics and increasing resistance research has started to focus on investigating the natural antibiotic resistome and understanding the ecology and evolution of antibiotic resistance in the non-clinical environment in order to identify reservoirs of both known and novel antibiotic resistance mechanisms.

Despite the belief that the soil antibiotic resistome bacteria play an increasingly important role in the evolution, development and spread of antibiotic resistance in humans and animals, there is little known about the natural bacterial resistome in soil. There have been many calls for more information about the natural resistome and these have also highlighted the importance of understanding the soil resistome in the preservation of antibiotics for the treatment of infections [1]–[4]. However, to date there have been few studies which have investigated the culturable soil resistome and these have been limited to the antibiotic producing bacteria *Streptomyces*, and an isolated cave microbiome [5], [6].

Culture based antibiotic susceptibility testing is the gold standard of antibiotic resistance testing in hospitals throughout the world. It is a relatively cheap and easy technique with little need for sophisticated or expensive equipment. Therefore, using susceptibility testing would enable the comparison of non-clinical data with clinical data. Antibiotic susceptibility testing also enables the phenotypic detection of as yet uncharacterized resistance mechanisms and complex resistance mechanisms such as efflux, which are frequently mediated by several genes. However, antibiotic resistance and antibiotic breakpoints have been defined within the context of their medical functions. Breakpoints define the thresholds of response of bacteria to an antibiotic [7]. Clinical breakpoints define bacteria as susceptible, intermediate or resistant to an antibiotic and are calculated using several factors, including clinical results from studies, antibiotic dosing and pharmacokinetic (PK) and pharmacodynamics (PD) measurements [8]. Breakpoints are used as a guide for the clinician to decide how to treat the patient, with antibiotic resistance meaning treatment failure. Clinical resistance is a complex concept in which the type of infecting bacterium, its location in the body, the distribution of the antibiotic in the body and its concentration at the site of infection, and the immune status of the patient all interact. The difficulty arises when we apply these definitions of resistance to soil bacteria or non-pathogenic bacteria, for which no breakpoints exist. Antibiotic resistant bacteria detected in soil to date have been defined as bacteria capable of growth at 20 µg/ml, as no breakpoints exist for the non-pathogenic bacteria found in soil [5], [6].

The bacteria that can be grown in the laboratory are only a small fraction of the total diversity that exists in nature. Approximately only 1% of bacteria on Earth can be readily cultivated *in vitro* [9], [10]. Therefore, non-culture based tools such as PCR and metagenomics are required to capture the non-culturable section of the non-clinical antibiotic resistome. However, these tools are limited to screening for known resistance genes and are not sufficient to characterize the intrinsic resistance or efflux resistance mechanisms, that are mediated by several genes.

Mechanistic commonalities between soil bacteria and clinical pathogens were first identified in the 1970s, including identical molecular aminoglycoside resistance mechanisms in *Streptomyces* and clinical pathogens [11]. However, the resistance mechanisms are not limited to antibiotic producing soil bacteria. The soil may be a reservoir of resistance genes, which are already present in human pathogens or which may emerge to increase the current arsenal of antibiotic resistance mechanisms in pathogens. Most antibiotics used in human medicine have been isolated from soil microorganisms. Therefore, soil is thought of as a potential reservoir of antibiotic resistance genes. The presence of antibiotics in soil is believed to have promoted the development of highly specific antibiotic resistance mechanisms in antibiotic producing and non-producing bacteria [4]. This belief is based on studies, which have identified resistance genes such as *bla*_CTX-M_, *qnrA* and *bla*_NDM_ as originating in the environmental bacteria *Kluyvera* sp., *Shewanella algae* and *Erythrobacter litoralis*, respectively [12]–[14]. These genes are clinically relevant resistance genes and are currently causing difficulties in the treatment of bacterial infections. The origins of other plasmid mediated resistance genes are still unknown. The commonly believed theory of the role of the soil resistome is based on the belief that antibiotic production and resistance co-exist in soil bacteria, as demonstrated by studies of antibiotic biosynthetic pathways and genome analysis [5], [15]. The theory relies upon the idea that without the resistance gene the antibiotic producing bacteria would self-destruct, on production of the antibiotic. However, Davies and Davies identified that this theory remains to be proven [16]. In order to understand the importance of soil as a potential reservoir of antibiotic resistance mechanisms we need to investigate the soil bacteria.

The soil is a reservoir of antibiotic resistance genes, but not all resistance mechanisms are necessarily a threat to the continued use of antibiotics in all pathogens [17]. Intrinsic resistance is a characteristic of almost all isolates of the bacterial species and occurs when the antimicrobial activity of the drug is clinically insufficient or antimicrobial resistance is innate, rendering it clinically ineffective [18]. The most intrinsically-resistant bacteria have a non-clinical origin (e.g. soil), which are less likely to have antibiotic selective pressures equivalent to hospitals. This suggests that the main physiological role of the elements involved in the intrinsic resistance phenotype is not conferring resistance to antibiotics [19], [20].

We aimed to identify the levels of culturable resistant bacteria, both antibiotic producers and non-antibiotic producers, and identify the roles of the different mechanisms of resistance such as efflux, novel enzymatic resistance mechanisms and selected plasmid mediated resistance genes in conferring multi-drug resistance within the soil bacterial community. We linked the bacterial phylogeny to the antibiotic resistance profiles in order to compare the intrinsic resistance profiles of soil and clinical bacteria from the same bacterial order.

## Results

**Figure 1:**
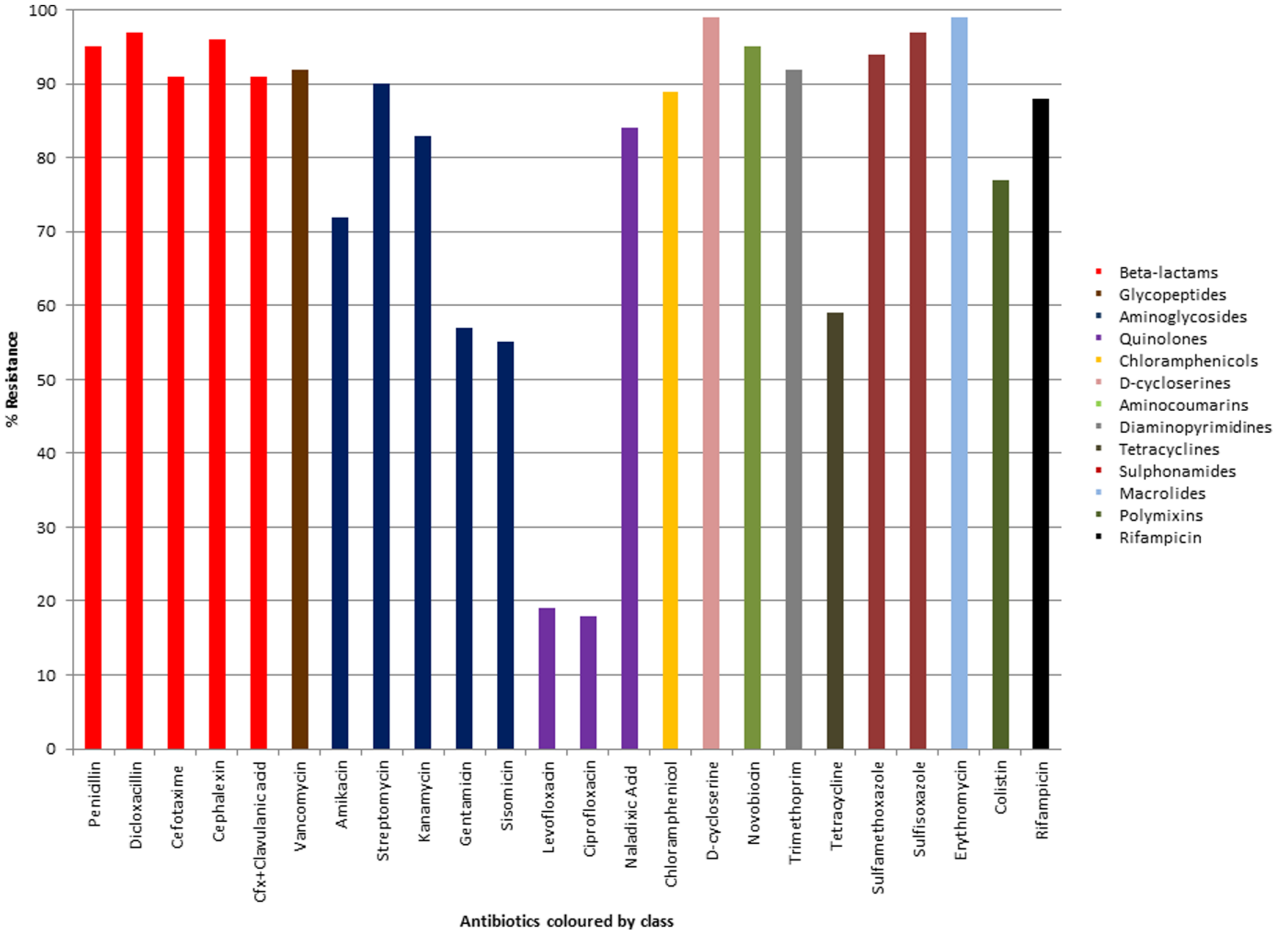
The antibiotic resistance profiles of all the study bacteria from ten soils. Isolates were individually screened for resistance to each of 23 antibiotics.

**Figure 2:**
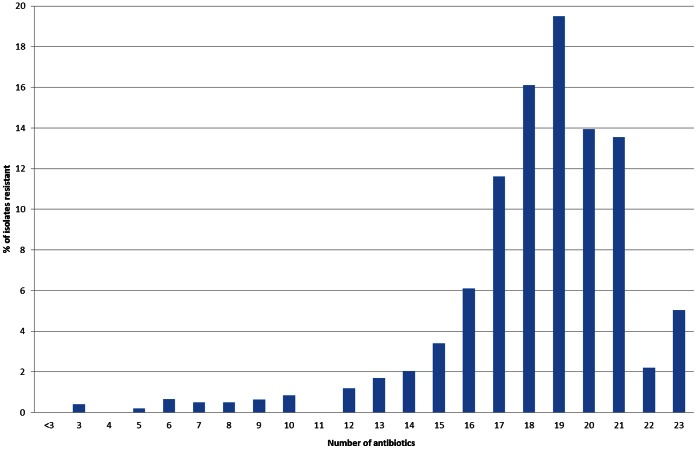
Resistance spectrum of the bacterial soil isolates. Antibiotic resistance was measured as growth on 20 µg/ml of antibiotic.

Four hundred and twelve antibiotic resistant bacterial isolates were cultured from ten soils from agricultural, urban and pristine environments (Table 1). All isolates were multi-drug resistant, of which greater than 80% were resistant to 16–23 antibiotics, comprising almost all classes of antibiotic (Figures 1, 2, S1, S2). The 23 antibiotics tested are used in agriculture, human and veterinary medicine and covered all known antibiotic classes and mechanisms of action. They included natural antibiotics such as penicillin and streptomycin, semi-synthetic antibiotics such as cefotaxime and cephalexin, synthetic antimicrobials such as ciprofloxacin and sulfamethizole, and antibiotics used as the last line of defense, vancomycin and colistin, to treat multi-drug resistant infections such as methicillin resistant *Staphylococcus aureus* (MRSA) and *Acinetobacter*, respectively. Multi-drug resistance is defined as resistance to three or more different classes of antibiotics.

**Table 1:** Geographical and descriptive characteristics of the analyzed soil samples.

Sample ID	Description	Environmental matter	Elevation (m)	Latitude	Longitude	pH
S1	Wädenswil Apple Orchard	Agriculture	407	47.2333	8.6667	7.0
S2	Einsiedeln	Urban	880	47.1167	8.75	7.1
S3	Hospital garden	Urban	667	46.7167	9.4333	7.3
S4	Lindau Apple Orchard	Agriculture	485	47.4833	8.2	4.1
S5	Güttingen Apple Orchard	Agriculture	503	47.6	9.2833	5.3
S6	Ruetli meadow	Pristine	835	46.9667	8.6	7.1
S7	Area beside Lake Zürich	Urban	408	47.3667	8.55	7.0
S8	Matterhorn mountain trail	Pristine	1936	46.0167	7.75	7.2
S9	Farmland treated with pig manure	Agriculture	592	47.1833	8.3167	5.7
S10	Mountain near Zürich	Urban	700	47.3496	8.492	7.0

Beta-lactam resistance ranged from 84% to 100% and was greater than 90% on average for the ten soils (Figure 1), indicating a natural high level of resistance to the β-lactams, both the natural penicillins and the semi-synthetic cephalosporin, in all soils and little variation between the soils. In contrast, an average of less than 20% of the soil populations were resistant to the fluoroquinolones; levofloxacin and ciprofloxacin, while individual soil resistance levels ranged from 0–48% (Figures 1, S3). However, resistance to the non-fluorine quinolone, nalidixic acid, was on average 84% and ranged from 65% to 97% (Figures 1, S3). Greater than 70% of the bacterial populations in all soils were resistant to the tetrahydrofolate synthesis inhibitor antibiotics; sulfisoxazole, sulfamethoxazole and trimethoprim and to rifampicin, vancomycin, chloramphenicol, D-cycloserine, novobiocin, and erythromycin. Resistance to the aminoglycosides; amikacin, streptomycin, kanamycin, gentamicin and sisomicin and to colistin and tetracycline were variable, dependent on the soil. Soils S2 (urban), S9 (agriculture) and S10 (urban) had higher levels of resistance to one or more aminoglycosides than the other soils and soils S4 (agriculture), S6 (pristine) and S7 (urban) (Figure S4) had lower resistance levels to tetracycline than the other soils (Figure S5). Colistin resistance varied from 34% in soil S6 (pristine) to 100% in soil S8 (pristine) (Figure S6). The variation in resistance levels were not due to the anthropogenic use of the soil.

**Figure 3:**
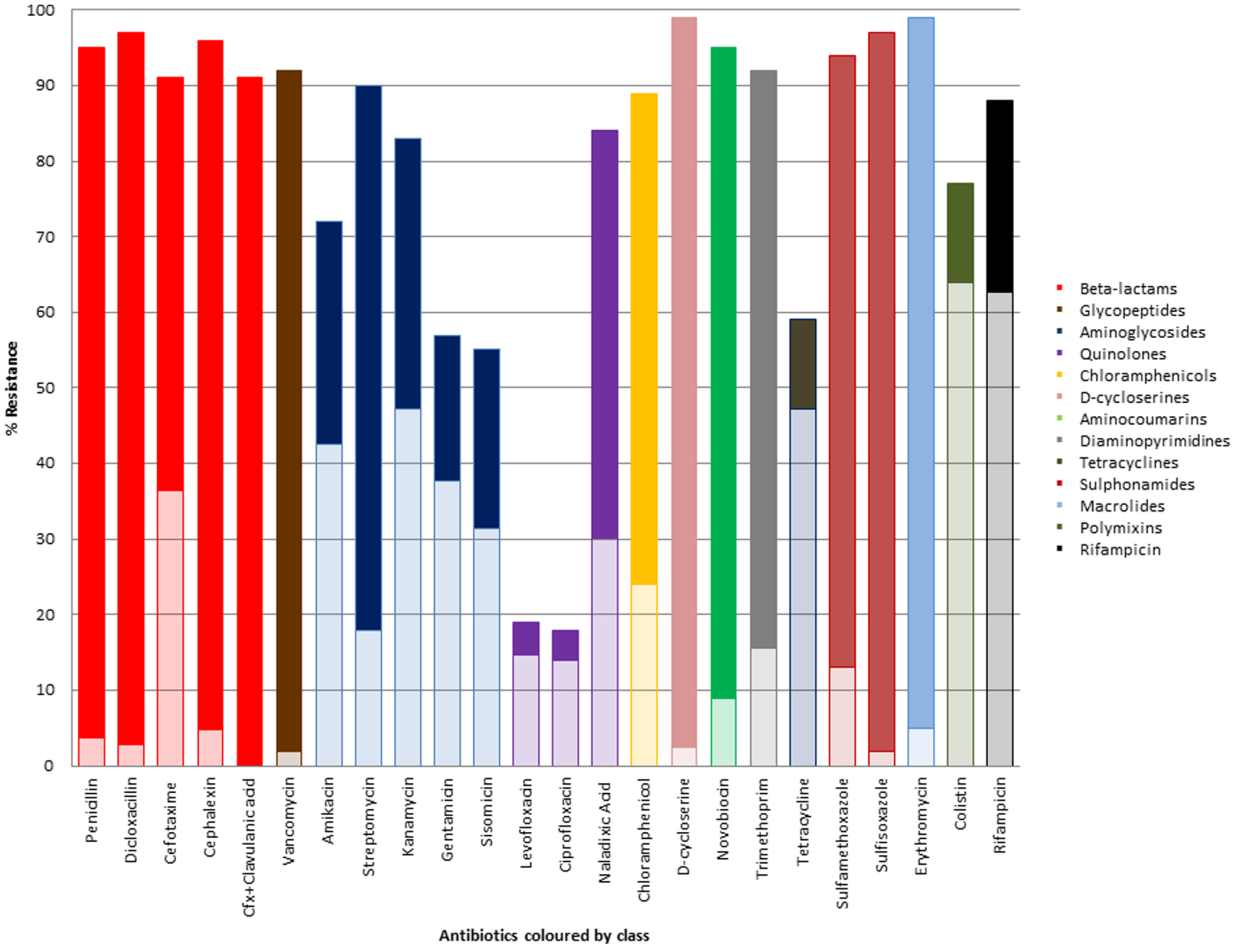
The antibiotic resistance profiles and percentage mediated by efflux of all the study bacteria. The antibiotics are color coded according to class. The total bar is the percentage resistance of all isolates to each antibiotic. The lightly shaded bar is the percentage of resistance mediated by efflux for each antibiotic.

Multi-drug resistance conferred by efflux was identified in 83% of the soil isolates and 410 of 412 isolates were capable of conferring resistance to at least one class of antibiotics by efflux. Over 76% of the fluoroquinolone (levofloxacin and ciprofloxacin) resistance was mediated by efflux (Figure 3). In comparison, efflux mediated resistance to nalidixic acid, the non-fluorine quinolone antibiotic, was identified in 36% of the resistant population. Efflux accounted for 80% and 72% of the resistance mechanisms to tetracycline and rifampicin, respectively and 83% of colistin resistance. Efflux was identified as the main mechanism of resistance to the aminoglycosides amikacin, gentamicin, sisomicin and kanamycin in greater than 56% of the total soil populations but only in 20% of streptomycin resistant isolates. Efflux mediated resistance to the β-lactams penicillin, dicloxacillin and cephalexin and to vancomycin was identified in very few isolates (≤5%). However, efflux mediated resistance to the cephalosporin cefotaxime was conferred in 40% of the resistant isolates. A low percentage of efflux mediated resistance was detected for the tetrahydrofolate reductase pathway inhibitors sulfamethoxazole (5%), sulfisoxazole (2%) and trimethoprim (17%). The antibiotics D-cycloserine, novobiocin and erythromycin also were also infrequently the substrates of efflux pumps. Whether the antibiotics were natural, semi-synthetic or synthetic did not play a role in the likelihood of them being a substrate for efflux.

**Figure 4:**
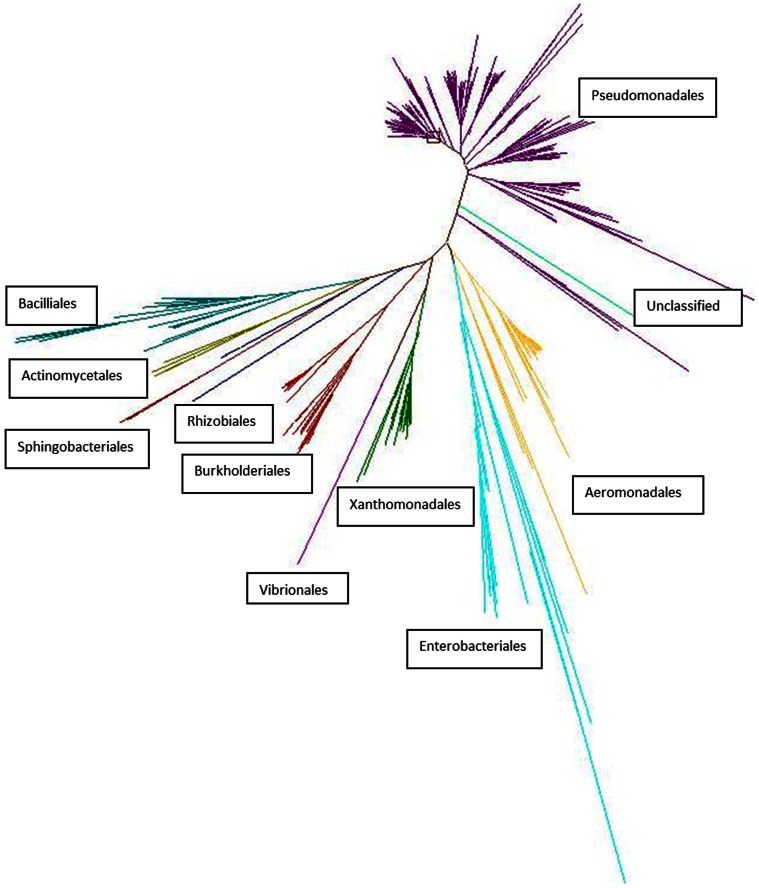
The phylogenetic distribution and relatedness of the soil bacteria. The 16S rRNA genes were sequenced from all bacteria and the phylogenetic tree was created using these sequences and the ARB program package. Branches of the tree are color coded by bacterial orders, with each line at the branch end representing an individual bacterial isolate. The orders with the largest number of lines indicate the largest number of bacterial members within this study. The relatedness of the orders is defined by the distance from one branch to the next. The unclassified bacteria are therefore, closest to the Pseudomonadales.

The phylogenetic profiling identified four phyla: Proteobacteria (81.7%), Firmicutes (6.3%), Actinobacteria (1.5%) and Bacteroidetes (1%) (Figure 4). The remaining 9.5% were unclassified bacteria. These phyla were repesented by ten orders including Pseudomonadales, Aeromonadales, Burkholderiales, Xanthomonadales, Bacillales, Enterobacteriales and Vibrionales. Over 50% of the isolates belonged to the order Pseduomonadales.

It is important to factor in the role of intrinsic resistance in the levels of resistance, especially to the β-lactams. The pathogenic bacteria *Pseudomonas aeruginosa*, *Stenotrophomonas maltophilia*, *Burkholderia cepacia*, *Acinetobacter* species, *Achromobacter xylosoxidans*, *Serratia marcescens* and *Aeromonas* species are intrinsically resistant to penicillin and the β-lactam antibiotics [18]. We defined intrinsic resistance as resistance of the majority of the population to the antibiotic as no definition exists for environmental bacteria. The Bacillales, Pseudomonadales, Burkholderiales, Xanthomonadales (*Stenotrophomonas* species), Enterobacteriales (78% *Serratia* species) and Aeromonadales constituted 87% of the soil bacterial population within this study. The classes of antibiotics used to treat *Pseudomonas* species infections include the fluoroquinolones, amikacin, gentamicin and colistin as an antibiotic of last resort. Intrinsic resistance to these antibiotics has not yet been identified in clinical *Pseudomonas* species and levels of resistance remains relatively low [22]. The dominant bacterial species investigated were intrinsically resistant to the β-lactams, which are most frequently constitutively or inducibly expressed β-lactamases. The results of the penicillin enzyme assay and the efflux tests confirmed that the resistance mechanisms were enzyme based and not efflux mediated, which concur with the intrinsic mechanisms identified in clinical bacteria from these orders.

In order to identify the impact of intrinsic resistance and efflux in the resistance profiles of the soil bacteria we separated the resistance levels according to most frequently identified bacterial orders (Table 2). Resistance to vancomycin, rifampicin, trimethoprim and erythromycin was mediated by intrinsic resistance or efflux in all six orders consistent with the intrinsic resistance of the clinically relevant species of these orders. Clinical isolates of *Burkholderia cepacia* and *Stenotrophomonas* are intrinsically resistant to amikacin and gentamicin, and *Serratia marcescens* to amikacin. The soil bacteria belonging to the orders Burkholderiales, Xanthomonadales (*Stenotrophomonas* species) and Enterobacteriales (almost all *Serratia* species) were also intrinsically resistant to the same aminoglycosides as their clinically relevant counterparts. In the soil bacterial populations the Bacillales, Pseudomonadales and the Aeromonadales were also intrinsically resistant to amikacin and gentamicin in contrast to clinical findings for the bacteria and the Enterobacteriales were additionally intrinsically resistant to gentamicin. Resistance to gentamicin was mainly mediated by efflux.

**Table 2:** The variations in the intrinsomes of soil and clinical bacteria.

Bacterial order	Penicillin(Soil/Clinical bacteria)	Amikacin(Soil/Clinical bacteria)	Gentamicin (Soil/Clinical bacteria)	Ciprofloxacin (Soil/Clinical bacteria)	Tetracycline (Soil/Clinical bacteria)	Colistin(Soil/Clinical bacteria)	Vancomycin (Soil/Clinical bacteria)	Rifampicin (Soil/Clinical bacteria)	Chloramphenicol (Soil/Clinical bacteria)	Trimethoprim(Soil/Clinical bacteria)
Bacillales	**I/N** a	**I/N**	**I/N**	**I/N**	N/N	**N/I**	I/I	**I/N**	**I/N**	**I/N**
Pseudomonadales	I/I	**I/N**	**I/N**	N/N	**I/N**	N/N	I/I	I/I	I/I	I/I
Burkholderiales	I/I	I/I	I/I	**N/I**	**I/N**	**N/I**	I/I	I/I	I/I	I/I
Xanthomonadales	I/I	I/I	I/I	**I/N**	**I/N**	**I/N**	I/I	I/I	**I/N**	I/I
Enterobacteriales (78%*Serratia* sp.)	I/I	I/I	**I/N**	N/N	**I/N**	**N/I**	I/I	I/I	N/N	**I/N**
Aeromonadales	I/I	**I/N**	**I/N**	N/N	N/N	**I/N**	**I/N**	I/I	N/N	**I/N**

The patterns of intrinsic resistance for each bacterial order are compared to those of their clinical counterparts. Those in bold highlight the variations in intrinsic resistance between soil bacteria of this study and their clinical counterparts.

a I = intrinsically resistant N = not intrinsically resistant.

Innate resistance to ciprofloxacin has been described only in the *Burkholderia cepacia* complex species of clinical bacteria [18]. However, in this study only ten of 34 Burkholderiales isolates were resistant to ciprofloxacin, nine of which were positive for efflux. In contrast to their clinically relevant species, the Xanthomonadales and Bacillales were both intrinsically resistant to ciprofloxacin. The remaining species had low levels of resistance to ciprofloxacin, mainly due to the non-specific efflux resistance mechanism.

Clinical *Pseudomonas aeruginosa* are intrinsically resistant to the tetracycline class [18]. However, the Burkholderiales, Xanthomonadales and Enterobacteriales, in addition to the Pseudomonadales, from the soils were also intrinsically resistant to tetracycline by efflux. Intrinsic resistance to colistin in clinical isolates has been described in gram-positive bacteria, such as Bacillales and the gram-negative *Burkholderia* and *Serratia marcescens* species. Our results identified that in contrast to the clinical findings, these species in the soil populations were not intrinsically resistant to colistin and the Xanthomonadales and Aeromonadales bacteria were intrinsically resistant to colistin and was mainly mediated by efflux in all species, except the Xanthomonadales.

The dissemination of extended spectrum β-lactamases (ESBL) and plasmid mediated quinolone resistance (PMQR) multi-drug resistant plasmids is causing increasing difficulty in the treatment and management of both community and hospital acquired infections. As these antibiotic resistance genes are thought to have their origins in environmental bacteria it was important to investigate the role of these genesin the soil resistome [11]–[14]. Extended spectrum β-lactamases, unlike AmpC β-lactamases, are inhibited *in vitro* by clavulanate. All β-lactam resistant isolates were tested for the presence of ESBL by comparison of growth on cefotaxime with growth on cefotaxime and clavulanate. Growth on cefotaxime but inhibition on cefotaxime and clavulanate suggested the presence of ESBL. Thirty isolates were ESBL positive, 50% of which were isolated from the urban soil S3. None of the plasmid mediated ESBL genes nor β-lactamase genes were detected in any of the isolates or the extracted soil DNA (Table 3). No inhibitor resistant β-lactamases were detected. The extracted DNA from the ten soil samples were screened for the presence of *bla*_NDM-1_, all samples were negative. Extended spectrum β-lactamase production was identified in a wide variety of orders: Enterobacteriales (6/18 bacterial isolates), Pseudomondales (6/221), Aeromonadales (5/31), Burkholderiales (5/34), unclassified (5/39), Actinomycetales (1/6), Bacilliales (1/26) and Xanthomonadales (1/28).

**Table 3:** Genotypic screening of mobile antibiotic resistance genes in the bacterial isolates and extracted soil DNA.

Antibiotic resistance phenotype	Study samples investigated	Resistance genes	Detected (n^b^)
ESBL^a^	Total soil DNA (n = 10) Bacteria isolates (n = 30)	*bla*_TEM_	0
ESBL^a^	Total soil DNA (n = 10) Bacteria isolates (n = 30)	*bla*_SHV_	0
ESBL^a^	Total soil DNA (n = 10) Bacteria isolates (n = 30)	*bla*_OXA,_	0
ESBL^a^	Total soil DNA (n = 10) Bacteria isolates (n = 30)	*bla*_CTX-M_ Groups 1, 2, 8, 9, 25	0
ESBL^a^	Total soil DNA (n = 10) Bacteria isolates (n = 30)	*bla*_VEB_	0
ESBL^a^	Total soil DNA (n = 10) Bacteria isolates (n = 30)	*bla*_PER_	0
ESBL^a^	Total soil DNA (n = 10) Bacteria isolates (n = 30)	*bla*_GES_	0
Quinolone	Total soil DNA (n = 10) Bacteria isolates (n = 346)	*aac(6′)Ib-cr*	0
Quinolone	Total soil DNA (n = 10) Bacteria isolates (n = 346)	*qnrA*	0
Quinolone	Total soil DNA (n = 10) Bacteria isolates (n = 346)	*qnrB*	0
Quinolone	Total soil DNA (n = 10) Bacteria isolates (n = 346)	*qnrS*	0
Quinolone	Total soil DNA (n = 10) Bacteria isolates (n = 346)	*qep*	0
Carbapenem	Total soil DNA (n = 10)	*bla*_NDM_	0

a ESBL = extended spectrum β-lactamase.

b n = number.

One hundred and four isolates resistant to the fluoroquinolones, levofloxacin and ciprofloxacin, and the total DNA extracted from the soils were screened for the presence of PMQR genes (Table 3). All fluoroquinolone resistant isolates and soil DNA extracted from the ten soils were negative for the *qnrA*, *qnrB*, *qnrS* and *qep* genes by PCR. All isolates were negative by sequencing for the *aac(6′)Ib-cr* gene. The DNA extracted from five soils contained novel *aac(6′)Ib* genes, with between two and seven amino acid mutations in comparison to the closest previously described protein or amino acid sequence in GenBank. These novel gene sequences have been deposited in GenBank (accession numbers KC916938, KC916939, KC916940). The DNA extracted from soils S1 (agriculture), S7 (urban) and S10 (urban) contained the same novel *aac(6′)Ib* gene (KC916938). Soils S4 (KC916939, agriculture) and S9 (KC916940, agriculture) each contained another novel *aac(6′)Ib* resistance gene containing two and three amino acid mutations, respectively, in comparison to their closest described amino acid sequence in GenBank.

**Figure 5:**
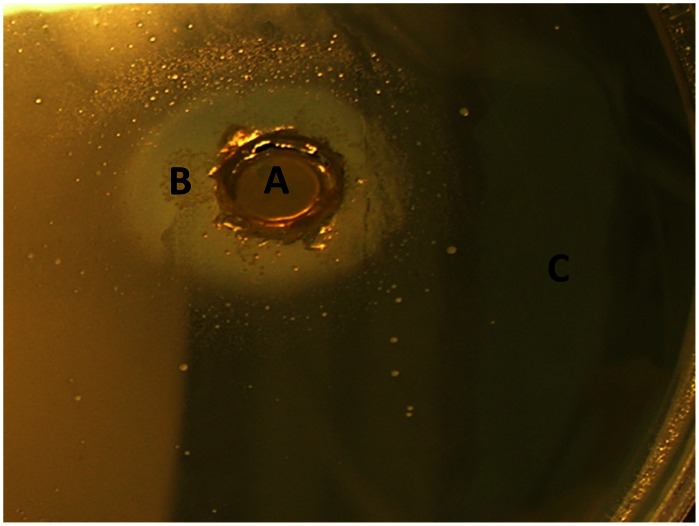
Enzyme inhibition assay using trimethoprim (20 µg/ml) and trimethoprim susceptible *S. aureus* ATCC 25923. An agar plate section containing trimethoprim (20 µg/ml), was inoculated with trimethoprim susceptible *S. aureus* ATCC 25923. An agar plug containing the trimethoprim inhibiting soil bacteria were placed into the inoculated trimethoprim agar. The soil bacteria producing the enzyme inhibiting trimethoprim is located in the center circle (A). The ring of growth of trimethoprim susceptible *S. aureus*, which has been protected from the inhibitory effects of trimethoprim by the soil bacteria enzyme (B). There is no *S. aureus* growth outside the zone of trimethoprim inhibition by the soil bacteria (C).

Antibiotic inactivation assays were performed in order to identify novel mechanisms of antibiotic resistance in the soil resistomes (Figure 5). Inactivation of penicillin and trimethoprim were identified in 100% and 20% of the investigated bacteria, respectively. The trimethoprim inactivating bacteria were investigated for inactivation of all other antibiotics assayed and did not result in inactivation of any except penicillin. Thus, this inactivation mechanism is a trimethoprim specific effect and not a non-specific effect, such as indole production [21]. The bacterial species containing the inactivation mechanism are indole negative. This is the first description of an inactivating resistance mechanism against trimethoprim. The principle of the assay was that antibiotic inactivating enzymes produced by the test bacteria would diffuse into the agar, in a similar fashion to antibiotics in the disk diffusion assay (Figure 5). None of the remaining antibiotic classes were inactivated using this assay, indicating that enzymatic inactivation of these classes of antibiotics within these bacteria was unlikely.

## Discussion

In order to analyze the soil antibiotic resistome, we investigated soils closest to human activities i.e. farming and urban, and compared these to a pristine soil population. While culture techniques will always bias the population of bacteria under study they have the advantage of being able to detect phenotypic resistances, including efflux mediated resistance and novel resistance mechanisms, and the resistance mechanism can be investigated in further detail. Culture techniques are also the gold standard for clinical testing of antibiotic resistance levels and certain resistance mechanism such as efflux or ESBL.

The resistance levels to almost all classes of antibiotics were extremely high. We identified the multi-drug resistant nature of soil bacteria, with greater than 80% of all isolates resistant to 16–23 antibiotics. Antibiotic resistance of bacterial pathogens consists of acquired resistance mechanisms and intrinsic resistance mechanisms. In order to elucidate the resistance mechanisms responsible for these high levels of resistance we investigated the bacteria and soil DNA for different resistance mechanisms, including efflux, production of ESBL and the presence of PMQR genes and antibiotic inactivation.

Intrinsic resistance in pathogenic bacteria has been well characterized in order to ensure that the most appropriate antibiotic therapy is administered [18]. In order to identify the role of the intrinsic resistance in the soil bacteria, the resistance phenotypes were linked to bacterial order. These were compared to the intrinsic resistances associated with the clinically relevant species of the same order (e.g., Pseudomonadales with *Pseudomonas aeruginosa*). We expected that the intrinsic resistance patterns of the clinically relevant species would correspond to the soil bacteria, in particular for bacterial pathogens that originated from soil, such as *Stenotrophomonas maltophilia*, *Burkholderia* species and *Pseudomonas* species. Resistance to the β-lactam and macrolide classes of antibiotics is mediated by intrinsic resistance in these clinically relevant species and soil bacteria. However, the pattern of intrinsic resistance of the soil bacteria of this study to the aminoglycosides, ciprofloxacin (for Burkholderiales only), tetracyclines, and colistin were not those associated with the corresponding pathogens. In some cases the soil bacteria were not intrinsically resistant (e.g., Burkholderiales to ciprofloxacin), and in others they were intrinsically resistant to antibiotics used to treat pathogenic bacteria (e.g., Pseudomonadales were intrinsically resistant to amikacin). *Burkholderia* species and *Stenotrophomonas maltophilia* are intrinsically resistant to all aminoglycosides, attributed to poor permeability and putative efflux [18]. These results suggest that while species such as *Pseudomonas* and *Stenotrophomonas* have their origins in soil, their closely related soil species do not necessarily contain the same intrinsic resistance mechanisms.

Multi-drug resistance was most frequently mediated by the efflux resistance mechanism and was identified in non-antibiotic producing bacteria. Resistance to colistin and tetracycline in particular was frequently mediated by efflux. Resistance to colistin in clinically relevant *Pseudomonas* species is most frequently associated with alterations in the outer membrane lipopolysaccharide (LPS) [25]. Efflux mediated colistin resistance has to date only been identified in *Yersinia* species but has not been well characterized [26]. Tetracycline resistance in Burkholderiales, Xanthomonadales, Enterobacteriales and Aeromonadales was most frequently mediated by efflux and in all case in greater than 50% of the resistant populations. Although efflux mediated resistance to tetracycline has been described in clinically relevant species it is infrequently identified in the populations. Intrinsic resistance (over 50% of the population resistant) to tetracycline in clinically relevant species of these orders has not been described. The identification of efflux mediated colistin and intrinsic tetracycline resistance in the soil bacteria identifies potential novel mechanisms of resistance, which could evolve in or be transferred to clinically relevant species over time.

Bacteria have the ability to survive in antibiotics due to their evolution in the presence of natural antibiotics over millennia but in our study this also holds true for the synthetic antibiotics, which have not been present in soils. These soil bacteria have developed or utilize universal mechanisms of resistance to overcome the inihibtion by natural antibiotics or toxins, which also work efficiently against many classes of antibiotics [27]. Efflux pumps are evolutionary ancient elements, highly relevant for the physiology and ecological behaviour of all living things [2]. However, efflux resistance is not confined to a chromosomal resistance mechanism but may be transferred on mobile elements, e.g. *qep* or recently a resistance-nodulation-cell division/multidrug resistance efflux mechanism [28], [29].

None of these ESBL or quinolone mediated plasmid mediated resistance genes were present in either the bacteria or the DNA. These results suggest that these resistance genes are either location or sample type specific or in extremely low numbers in the environment. The lack of *bla*_NDM_ β-lactamases in the soil DNA is similar to findings from a study conducted in another European site (Cardiff wastewater treatment plant), which also contained no *bla*_NDM_ genes but are in contrast to environmental sites in New Delhi, India, where *bla*_NDM_ was detected in two of 50 drinking-water samples and 51 of 171 seepage samples [23]. The first *bla*_NDM-1_ infections in Switzerland were isolated from three patients in Geneva university hospitals between 2009 and 2010 [24]. Thus, it is unlikely that Swiss soils were the sources of these clinical *bla*_NDM_ positive bacteria.

This study identified the first inactivating resistance mechanism against trimethoprim in 21 different bacterial isolates from five different soils, comprising farmland and urban environments. Although previous studies, restricted to cave bacteria and Actinomycetes, have investigated soil bacteria for trimethoprim inactivating enzymes they did not result in their identification [5], [6]. Trimethoprim is a synthetic antibiotic, which is not used in agriculture and thus is restricted to clinical use and it is unlikely that the soil bacteria were exposed to trimethoprim. The presence of an inactivation mechanism against a synthetic antibiotic suggests that it may have evolved for an alternative function. The trimethoprim inactivation mechanism was identified as trimethoprim specific, as these bacteria did not inactivate any other class of antibiotics. Penicillin inactivating enzymes were also identified in all investigated resistant isolates. Gram-negative bacteria frequently contain chromosomally mediated β-lactamases and have most likely developed these mechanisms through millennia of co-evolution with penicillin producing soil dwelling fungi. High levels of β-lactam inactivation were identified in penicillin resistant bacteria isolated from an isolated cave [6]. Thus, the high frequency of penicillin inactivating enzymes is due to intrinsic resistance of these bacteria.

There have been many calls for more information about the natural resistome and these have also highlighted the importance of understanding the soil resistome in the preservation of antibiotics for the treatment of infections [3], [4], [27]. Our study provided a comprehensive description of the soil resistome in relation to multi-drug resistance together with phylogenetic analysis of culturable soil bacteria under a range of anthropogenic influences. The novel inactivation mechanism detected in this study suggest that the soil bacteria could be a reservoir of resistance mechanisms to synthetic antibiotics as well as natural antibiotics.

While soil bacteria are naturally resistant to many different classes of antibiotics, natural, snythetic and semi-synthetic, the resistance mechanisms are most frequently the non-specific efflux or intrinsic resistance mechanisms. The efflux mechanisms could be present on mobile elements or may be transferred to mobile DNA and cause increased difficulties in the treatment of bacterial pathogens. We have identified that the patterns of intrinsic resistance of the soil bacteria differ from the clinical isolates of the same phyla. Clinical isolates of Burkholderiales are intrinsically resistant to ciprofloxacin and colistin but soil isolates of the order Burkholderiales were not intrinsically resistant to ciprofloxacin or colistin. Similarly, clinical isolates of *Serratia marcescens* and *Bacillus* species are intrinscially resistant to colistin but were not intrinscially resistant to colistin in the soil population. The results of this study identified that the intrinsic resisome of soil bacteria is in contrast to that of their closely related clinically relevant species. These results suggest that the evolution of intrinsic resistance in soil and clinical bacteria has not developed along the same lines, although there may be similarities in certain resistance genes detected in both environments.

The conclusions of this study are that there was a high level of multi-drug resistance in soil bacteria to a wide variety of antibiotics and was not dependent on the soil use. This MDR was most frequently conferred by efflux, which if present on or transferred to mobile elements could cause increased difficulties in the treatment of human bacterial pathogens. These results can be used to enhance the understanding of the emergence and dissemination of novel antibiotic resistance from the natural reservoir to the clinical setting, which may aid the development of inhibitors of resistance mechanisms and resistant bacteria.

## Methods

### Antibiotics

The following antibiotics and antibiotic resistance mechanism inhibitors were used in this study: Penicillin, dicloxacillin, amikacin, cephalexin, kanamycin (Carl Roth GMBH and CO. KG), gentamicin, sisomicin, streptomycin, vancomycin (Carl Roth GMBH and CO. KG), levofloxacin, ciprofloxacin, sulfamethizole, nalidixic acid, chloramphenicol, d-cycloserine, cefotaxime, novobiocin, trimethoprim, sulfisoxazole, tetracycline, erythromycin, colistin, rifampicin, cefotaxime, clavulanic acid, phenylboronic acid and carbonyl cyanide 3-chlorophenylhydrazone (CCCP). All antibiotics and chemicals, except kanamycin and vancomycin, were obtained from Sigma-Aldrich Chemie Gmbh, Buchs SG, Switzerland. All antibiotic solutions were prepared according to manufacturer’s instructions.

### Description of Soils

The soils were collected from four agricultural sites, four urban sites and two pristine environments as described in Table 1. No specific permissions were required for these locations, as they were part of the research centers field trials or were not protected land. The field studies did not involve endangered or protected species.

### Culturing of Antibiotic Resistant Soil Bacteria

The antibiotic resistant bacteria were isolated as previously described [5]. Soil samples (1.9 g) were suspended in 15 ml LB Broth. The solution was shaken and allowed to settle for 5 minutes. Aliquots of 500 µl from supernatants of each soil suspension were inoculated into 4.5 ml of LB broth in Ritter Riplates® (Ritter GmbH, Schwabmuenchen, Germany) and incubated at 22°C for 3 days. Aliquots of 200 µl from each soil sample culture was transferred to 1.8 ml LB broth containing 20 µg/ml of each antibiotic and incubated at 22°C for 4 days. The cultures were serially diluted on LB plates containing 20 µg/ml of the corresponding antibiotic and incubated at 22°C for 48 h. The individual colonies were plated onto antibiotic LB agar plates to obtain pure cultures. µg/ml.

### Antibiotic Resistance Profiling

The antibiotic susceptibilities were investigated as previously described [5]. Four hundred and twelve isolates representing the antibiotic resistance populations of the ten soils were isolated. Each isolate was inoculated into 200 µl LB in 96 well plates from frozen glycerol stocks and incubated at 22°C for 3 days. LB plates, each containing 1 antibiotic at 20 µg/ml, were inoculated using a multipoint inoculator. LB agar plates without antibiotic were used as controls. The inoculated plates were incubated at 22°C for 4 days. Bacteria which presented visible growth on agar plates containing 20 µg/ml of antibiotic were defined as resistant [5].

Twenty three different antibiotics were tested. The ESBL phenotype was determined by resistance to cefotaxime and susceptibility to cefotaxime (20 µg/ml) with clavulanic acid (4 µg/ml). Phenotypic efflux resistance was defined as resistance to the antibiotic and susceptibility to the antibiotic when carbonyl cyanide 3-chlorophenylhydrazone (CCCP) at 20 µg/ml was added to the agar. Growth of 152 isolates were inhibited by CCCP at 20 µg/ml. Inhibition of these isolates by CCCP were tested at doubling dilution concentrations of 0.5 µg/ml to 10 µg/ml. These isolates were not inhibited by CCCP alone at a final concentration of 1 µg/ml. Therefore, efflux in these isolates was defined as resistance to an antibiotic and susceptibility to the antibiotic when 1 µg/ml CCCP was added.

### Total Soil DNA Extraction

The total soil DNA from each of the ten soils was extracted using the Mo Bio PowerSoil® DNA Isolation Kit, Mo Bio Laboratories Inc. (Süd-Laborbedarf GmbH, Gauting, Germany).

### Antibiotic Resistance Gene Screening

Phenotypically ESBL positive isolates and the extracted total DNA from the ten soils were screened by PCR for the *bla_TEM_*, *bla_SHV_*, *bla_OXA_, bla*_CTX-M_ Groups 1, 2, 8, 9 and 25, *bla_VEB_*, *bla_PER_*, *bla_GES_* ESBL resistance genes [30]–[32]. The fluoroquinolone resistant isolates and the extracted total DNA from the ten soils were screened by PCR for the *aac(6′)Ib-cr*, *qnrA*, *qnrB*, *qnrS* and *qep* resistance genes using previously described primers and parameters [28], [33], [34]. *aac(6′)Ib-cr* PCR products were sequenced using the PCR primers. The DNA samples extracted directly from the soils were additionally screened for the presence of *bla_NDM-1_*. The *bla_NDM-1_* PCR primers were NDMF 5′ GAAGCTGAGCACCGCATTAG 3′ and NDMR 5′ TGCGGGCCGTATGAGTGATT 3′. The annealing temperature was 55°C and the PCR product of the positive control was approximately 800 bp. All resulting PCR products were sequenced. Positive controls were included in each PCR run except for *bla_VEB_*, *bla_PER_*, *bla_GES_*.

All new data has been deposited in GenBank with the accession numbers KC916938, KC916939, KC916940.

### Phylogenetic Profiling

The 16S ribosomal RNA (rRNA) genes of all isolates were amplified using bacterial 16S primers: Bact_63f 5′ CAGGCCTAACACATGCAAGTC 3′ and Bact_1389r 5′ ACGGGCGGTGTGTACAAG 3′ [35]. The resulting PCR product was on average 1363 bp. For the bacterial isolates which produced no PCR product with these primers a second primer set was utilised [36]: 16SF 5′ TCCTACGGGAGGCAGCAGT 3′ and 16SR 5′ GGACTACCAGGGTATCTAATCCTGTT 3′. 16S rRNA gene amplicons were sequenced and classified using Greengenes [37]. Phylogenetic trees were constructed using the neighbor-joining algorithm in ARB using the Greengenes aligned 16S rRNA gene database [38].

### Enzyme Inhibition Assays

The enzyme inhibition assays were performed using a plug assay. *Escherichia coli* ATCC 25922 or *Staphylococcus aureus* ATCC 25923 inocula were prepared to a 0.5 McFarland standard and 80 µl was spread on LB agar plates containing 20 µg/ml of penicillin, cefotaxime, kanamycin, tetracycline, gentamicin, amikacin, colistin, ciprofloxacin, levofloxacin, nalidixic acid, trimethoprim, rifampicin, erythromycin, vancomycin, streptomycin or chloramphenicol. Wells were cut aseptically in the antibiotic agar. Plugs corresponding to the same diameter as the wells were cut aseptically from the resistant bacteria plates and inserted into the wells of antibiotic containing plates. The plates were incubated at 37°C for 1–2 days. A circle of growth around the plugs, which reduced in cell density in relation to its distance from the plug, indicated antibiotic inactivation.

## Supporting Information

Figure S1 **The antibiotic resistance profiles segregated according to soil for all antibiotics.** The antibiotics are color coded according to class.
(TIF)

Figure S2 **The numbers of antibiotics each soil bacterial community is resistant to as a percentage of the total bacterial popoulation within the given soil.**
(TIF)

Figure S3 **The quinolone antibiotic resistance profiles of the soil bacteria separated according to soil sample.**
(TIF)

Figure S4 **The aminoglycosides antibiotic resistance profiles of the soil bacteria separated according to soil sample.**
(TIF)

Figure S5 **The tetracycline antibiotic resistance profiles of the soil bacteria separated according to soil sample.**
(TIF)

Figure S6 **The colistin antibiotic resistance profiles of the soil bacteria separated according to soil sample.**
(TIF)
