# Supplementary figures and images for: The Culturable Soil Antibiotic Resistome: A Community of Multi-Drug Resistant Bacteria
Source: PLoS One. 2013 Jun 12;8(6):e65567. doi: 10.1371/journal.pone.0065567 (PMC3680443; doi:10.1371/journal.pone.0065567)

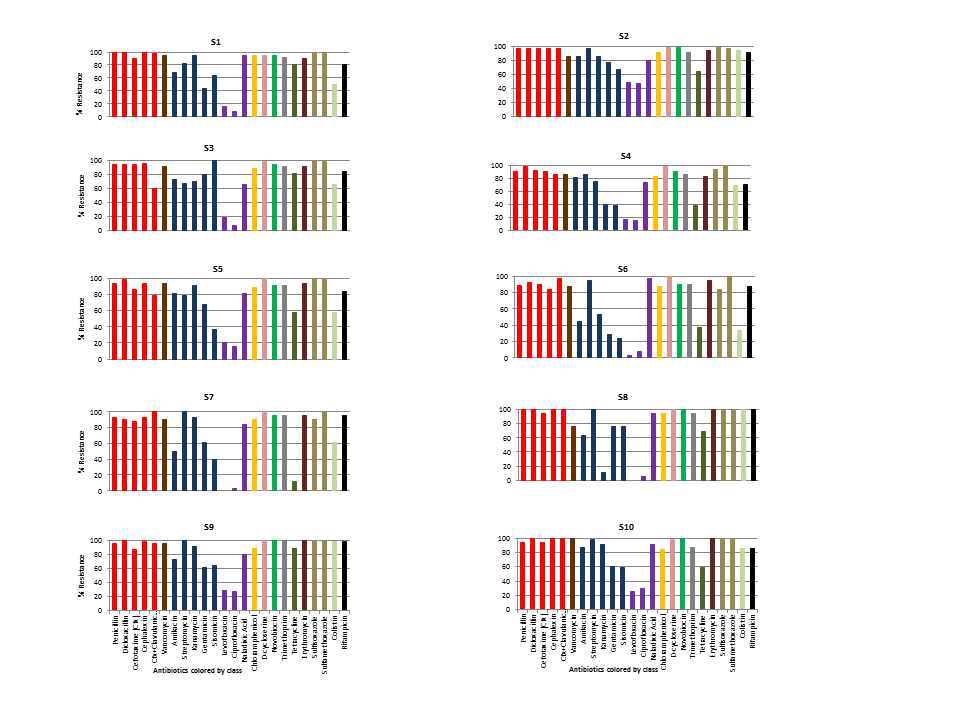

Supplement: Figure S1 — The antibiotic resistance profiles segregated according to soil for all antibiotics. The antibiotics are color coded according to class. (TIF) [file pone.0065567.s001.tif]

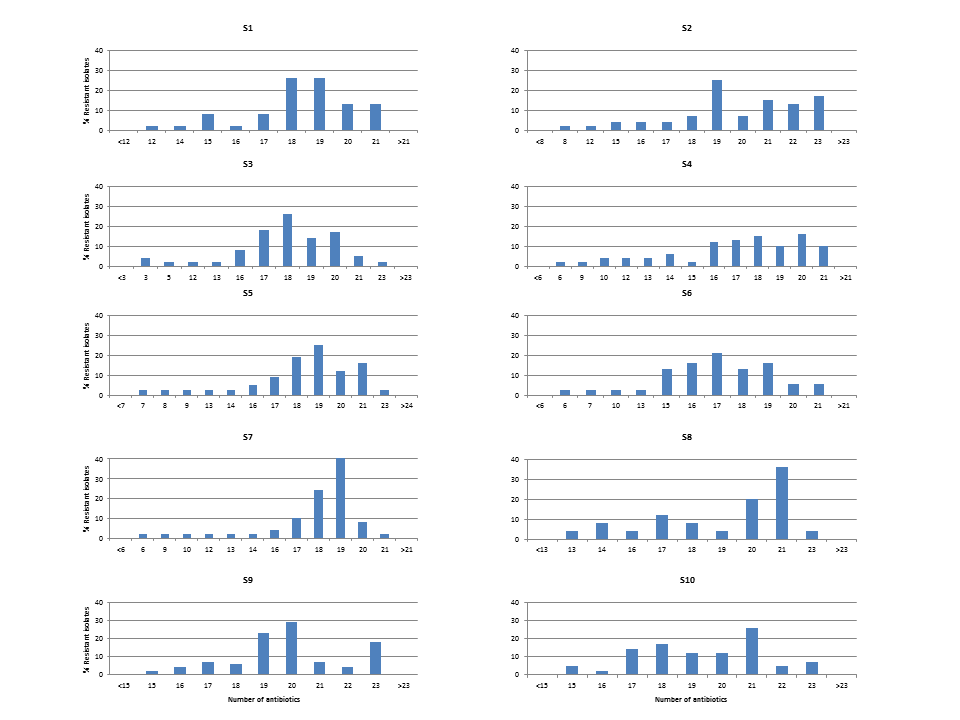

Supplement: Figure S2 — The numbers of antibiotics each soil bacterial community is resistant to as a percentage of the total bacterial popoulation within the given soil. (TIF) [file pone.0065567.s002.tif]

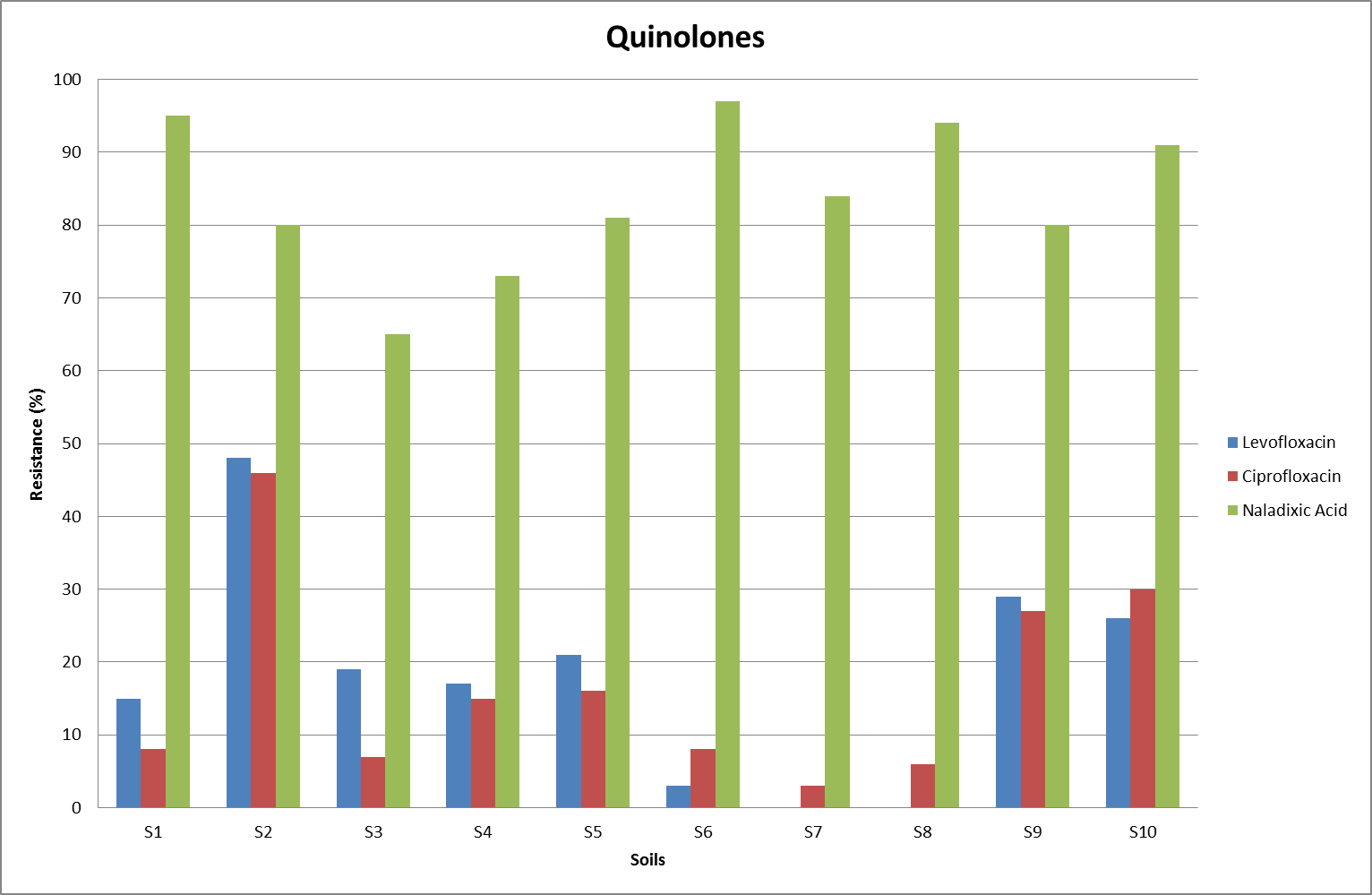

Supplement: Figure S3 — The quinolone antibiotic resistance profiles of the soil bacteria separated according to soil sample. (TIF) [file pone.0065567.s003.tif]

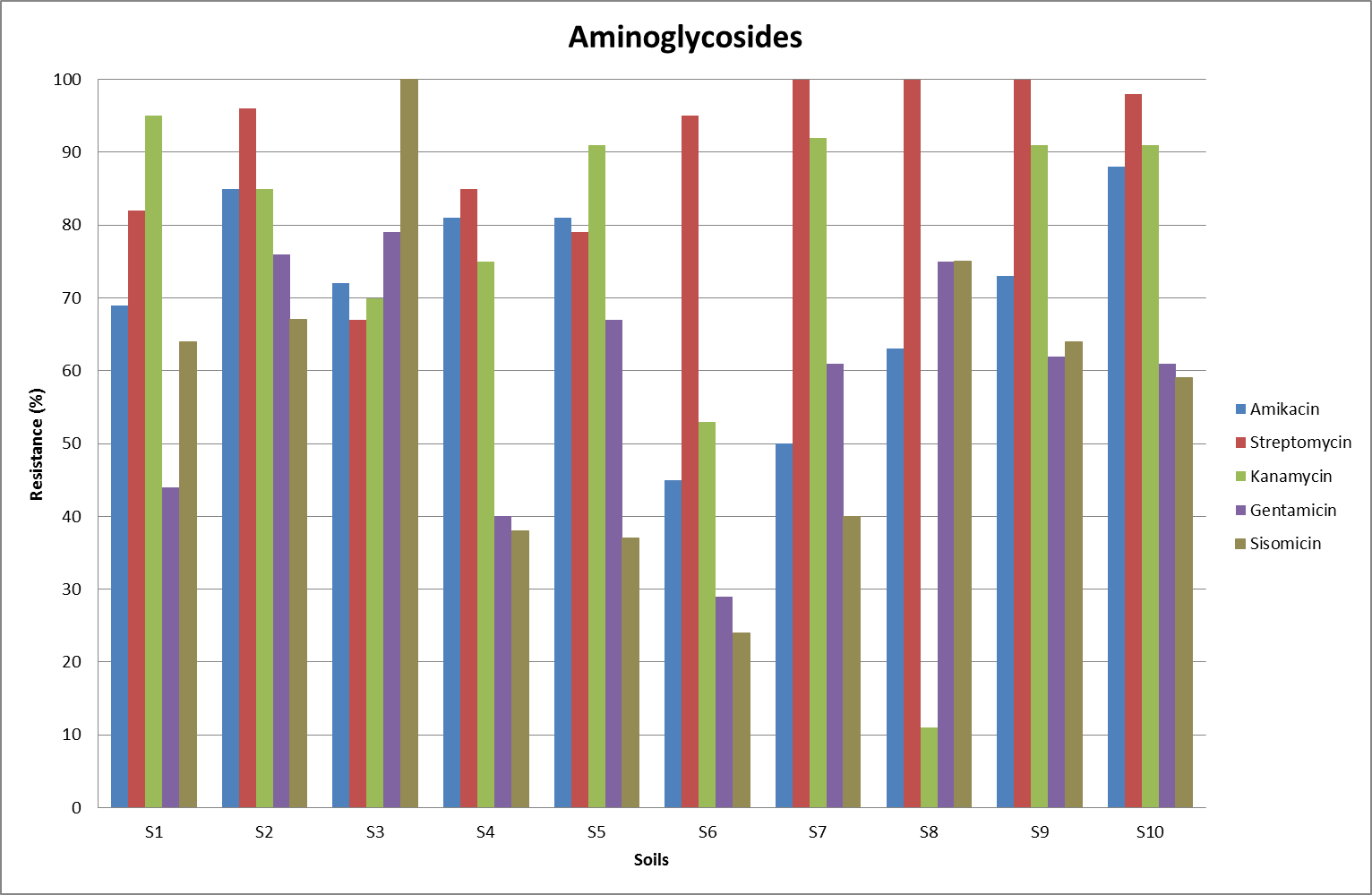

Supplement: Figure S4 — The aminoglycosides antibiotic resistance profiles of the soil bacteria separated according to soil sample. (TIF) [file pone.0065567.s004.tif]
